# Production, characterization, and application of *Pseudoxanthomonas taiwanensis* biosurfactant: a green chemical for microbial enhanced oil recovery (MEOR)

**DOI:** 10.1038/s41598-024-61096-1

**Published:** 2024-05-04

**Authors:** Isty Adhitya Purwasena, Maghfirotul Amaniyah, Dea Indriani Astuti, Yoga Firmansyah, Yuichi Sugai

**Affiliations:** 1 Microbiology Study Program, School of Life Sciences and Technology, Bandung Institute of Technology, Ganesha No 10, Bandung, West Java 40132 Indonesia; 2 Politeknik Negeri Banyuwangi, Livestock Product Processing Technology Study Program, Jl. Raya Jember Km. 13, Labanasem, Kabat, Banyuwangi, East Java 68461 Indonesia; 3 Department of Earth Resources Engineering, Faculty of Engineering, Kyushu University, 744 Motooka, Nishi-ku, Fukuoka, 819-0395 Japan

**Keywords:** *Pseudoxanthomonas taiwanensis*, Biosurfactant, Core flooding, Glycolipid, MEOR, Applied microbiology, Environmental microbiology

## Abstract

Biosurfactants, as microbial bioproducts, have significant potential in the field of microbial enhanced oil recovery (MEOR). Biosurfactants are microbial bioproducts with the potential to reduce the interfacial tension (IFT) between crude oil and water, thus enhancing oil recovery. This study aims to investigate the production and characterization of biosurfactants and evaluate their effectiveness in increasing oil recovery. *Pseudoxanthomonas taiwanensis* was cultured on SMSS medium to produce biosurfactants. Crude oil was found to be the most effective carbon source for biosurfactant production. The biosurfactants exhibited comparable activity to sodium dodecyl sulfate (SDS) at a concentration of 400 ppm in reducing IFT. It was characterized as glycolipids, showing stability in emulsions at high temperatures (up to 120 °C), pH levels ranging from 3 to 9, and NaCl concentrations up to 10% (w/v). Response surface methodology revealed the optimized conditions for the most stable biosurfactants (pH 7, temperature of 40 °C, and salinity of 2%), resulting in an EI24 value of 64.45%. Experimental evaluations included sand pack column and core flooding studies, which demonstrated additional oil recovery of 36.04% and 12.92%, respectively. These results indicate the potential application of *P. taiwanensis* biosurfactants as sustainable and environmentally friendly approaches to enhance oil recovery in MEOR processes.

## Introduction

Enhanced oil recovery (EOR) has garnered substantial attention in the oil and gas industry due to various compelling factors. The demand for energy continues to rise globally, while conventional oil recovery methods have shown limitations in efficiently extracting the available oil reserves^1–3^. Conventional recovery methods, primarily relying on gas pressure and natural reservoir forces, can only access approximately one-third of the oil present in known reservoirs. This leaves a significant portion of oil, ranging from 35 to 55%^4,5^, trapped within reservoirs due to challenges such as high viscosity and interfacial tension differences between oil and water^6^.

To address these limitations and enhance oil recovery, tertiary methods like chemical enhanced oil recovery (CEOR) have gained prominence^7,8^. Among the CEOR techniques, microbial enhanced oil recovery (MEOR) has emerged as a sustainable and innovative approach. MEOR leverages microbial activities and their metabolic byproducts to improve oil extraction from depleted and marginal reservoirs^9,10^. By utilizing low-cost substrates and raw materials, MEOR produces valuable, biodegradable, and environmentally friendly chemical products, positioning them as green alternatives^11^. This integrated approach contributes to the maximization of oil reserve recovery, extending the lifespan of fields, and increasing the recovery factor of wells^12^.

Biosurfactants play a pivotal role within the realm of MEOR technology as surface-active substances. These molecules, produced by diverse microorganisms, come in several categories based on their chemical structure. Notable categories include glycolipids, lipopeptides, phospholipids, polymeric, and particulate biosurfactants^13^. Among these, lipopeptides (e.g., surfactins, lichenysins, fengycins) and glycolipids (e.g., rhamnolipids, sophorolipids, trehalolipids) stand out as the most commonly utilized in MEOR applications^14^. Compared to chemically produced surfactants, biosurfactants offer several advantages. They are inherently environmentally friendly, non-toxic, and biodegradable, making them ideal for sustainable EOR practices. Additionally, biosurfactants demonstrate remarkable effectiveness across a wide range of extreme conditions, including variations in temperature, pH, and salinity. These attributes make them versatile and adaptable to the diverse environments encountered in oil reservoirs^14–16^.

Implementing biosurfactants in MEOR can occur through two principal approaches: in-situ and ex-situ^17^. In the in-situ strategy, biosurfactant production takes place within the reservoir by stimulating the growth of indigenous bacteria through nutrient injection or by introducing specific bacteria strains. Conversely, ex-situ strategies involve biosurfactant production in controlled bioreactors, followed by the injection of the extracted biosurfactant into the reservoir via an injection well. Ex-situ strategies offer the advantage of not relying on the metabolic activity of bacterial strains within the reservoir, making them suitable for application in various types of reservoirs.

While the majority of biosurfactant-related MEOR studies have primarily focused on their ability to reduce interfacial tension (IFT) between crude oil and water^18^, recent research has broadened the scope. Emulsification activity and wettability alteration, mediated by biosurfactants, have gained increasing attention for their significant role in enhancing oil recovery during the EOR process^19^. Biosurfactants facilitate the release of trapped oil in reservoir rock pores, improving the mobility ratio between water and oil^20^. In ex-situ MEOR applications, a substantial quantity of biosurfactant is typically required.

The choice of carbon sources for biosurfactant production has a notable impact on the process, influencing properties such as yield, surface tension, viscosity, emulsification properties, molecular weight, and thermo-stability. However, it’s important to emphasize that different carbon sources generally do not significantly alter the chemical structure of the biosurfactant^21,22^. Therefore, optimizing carbon sources is critical to achieving the desired properties and maximizing the effectiveness of biosurfactants in ex-situ MEOR applications.

This study builds upon previous research by investigating the biosurfactant production capabilities of *P. taiwanensis* bacteria^23^. Four distinct carbon sources were meticulously examined to determine the most effective medium for biosurfactant production. Subsequently, the chemical structure of the biosurfactant was thoroughly characterized. Furthermore, the biosurfactant’s impact on wettability alteration and emulsification activity was assessed through contact angle measurements and emulsion determination experiments. These experiments considered various environmental variables such as pH, temperature, and salinity, utilizing response surface methodology (RSM) to provide comprehensive insights. To evaluate the practical applicability of the biosurfactant in MEOR, core flooding experiments were conducted. In these experiments, biosurfactant is injected into reservoir core samples to replicate actual conditions in oil well. To the best of our knowledge, this investigation is the first evidence reporting the characterization and potential utilization of the glycolipid-type biosurfactant produced by *P. taiwanensis* in EOR process by using core flooding experiment. The primary goal of this study is to build a strong foundation for advancing biosurfactants in MEOR, which also aims to promote the use of ecologically benign chemicals in EOR processes and increase the efficiency of oil extraction.

## Materials and methods

### Microorganism

Thermophilic bacteria, specifically *P. taiwanensis*, were isolated from an oil reservoir^23^. Based on its morphological characteristics, this Gram-negative bacterium is rod-shaped and aerobic, has an entire margin and exhibits a light-yellow color. To preserve these bacteria, stone salt mineral solution (SMSS) agar plates supplemented with 1% crude oil were utilized. The SMSS medium contains the following components per liter: NH_4_NO_3_ (2.5 g), MgSO_4_·7H_2_O (0.5 g), MnCl_2_·4H_2_O (0.2 g), CaCO_3_ (0.5 g), Na_2_HPO_4_·7H_2_O (1 g), KH2PO4 (0.5 g), and FeSO4·7H_2_O (0.1 g). Additionally, trace elements were added to the medium (g/L): CuSO_4_·7H_2_O (0.001 g), H_3_BO_3_ (0.03 g), COCl_2_ (0.02 g), ZnSO_4_·7H2O (0.01 g), and MnO_4_ (0.006 g). Crude oil was selected as the carbon source for biosurfactant production since the bacteria were isolated from an oil reservoir, and the product is intended to be injected back into oil reservoirs for MEOR applications. Subsequent subculturing was performed on Nutrient agar plates, which served as the inoculum for biosurfactant production.

### Selection of carbon source for biosurfactant production

Initially, various carbon sources were explored to optimize biosurfactant production, including 4% glucose, 4% molasses, 4% glycerol, and 2% crude oil. The crude oil used in this study was sourced from an Indonesian oil reservoir and had a specific gravity of 22–28°API, a density of 0.9098 g/mL at 25 °C, and a viscosity of 58.9 cP. The SMSS medium used for the enrichment culture of biosurfactant production. The pH of the medium was adjusted to 7.0, and the cultures were inoculated with 10% of a 24-h inoculum. Incubation was carried out at 50 °C and 120 rpm for 84 h. Following incubation, the culture was centrifuged at 7500 rpm for 30 min, yielding a cell-free supernatant (CFS) containing the biosurfactant for subsequent analytical measurements. While crude oil was employed as a carbon source, it was separated from the SMSS medium using Whatman No.2 filter paper. As a control, SMSS medium without a carbon source was also prepared. The selection of the best carbon source for biosurfactant production was based on parameters such as interfacial tension (IFT) and emulsification index (EI24) between CFS and crude oil.

### Growth profile, biosurfactant yield, Interfacial tension and emulsification activity

*Pseudoxanthomonas taiwanensis* was cultivated in the biosurfactant production medium (SMSS) and incubated at 50 °C and 120 rpm for a duration of 96 h. Bacterial growth was assessed using the total plate count method. The CFS was collected at regular 12-h intervals to investigate biosurfactant production, IFT, and EI24. To quantify the biosurfactant, crude biosurfactant was extracted from the CFS using purification techniques, and its quantity was determined as the amount of raw biosurfactant obtained per gram of production medium volume, following the method described by Satpute et al.^24^. Emulsification activity was evaluated by calculating the emulsification index, as originally described by Cooper and Goldenberg^25^ with modifications. Interfacial tension (IFT) between crude oil and the CFS was measured either using DropMaster DMs-401 or Tensiometer Du Nuoy.

### Characterization of biosurfactant produced* by P. taiwanensis*

#### Efficiency of CFS activity

The efficiency of the biosurfactant solution was evaluated by measuring the IFT of CFS at various concentrations (100%, 50%, and 10%), with water serving as a control. Additionally, a comparison was conducted between the IFT values of the CFS and sodium dodecyl sulfate (SDS) at different concentrations (200, 400, 600, 800, and 1000 ppm) to determine the extent of IFT reduction achieved by the biosurfactant in comparison to the synthetic surfactant.

#### Effect of the biosurfactant on contact angle

The contact angle of the CFS was measured using the sessile drop method with the DropMaster DMs-401 instrument. To perform the measurement, a glass plate was coated with a layer of crude oil on its surface. A drop of the crude biosurfactant was then carefully placed onto the oil-coated plate. In addition, the contact angle of water was measured as a point of comparison in the experiment using the same instrument^26^. The contact angle measurements were repeated at least three times for each sample to ensure accuracy and reproducibility, and the average values were reported.

#### Heavy oil mobility test

A crude oil mobility test was conducted on a glass plate, following a methodology similar to that described by Roldán-Carrillo et al.^27^, with some modifications. In this test, 1 mL of heavy oil samples with a specific gravity of 19° API was mixed with 1 mL of SMSS medium, 1 mL of CFS, and 1 mL of a crude biosurfactant solution in separate tubes. Subsequently, 1 mL of each mixture was carefully dropped onto the glass plate, and the distances traveled by the oil were measured after the displacement occurred. This procedure was repeated for multiple trials, and the results were reported as the average distance traveled by the oil.

#### Determination of critical micelle concentration (CMC)

The CMC value was determined by measuring the IFT of the crude biosurfactant stock solution at different concentrations (1.92; 1.95; 1.98; 2.01; and 2.04 g/L) into deionized water at a temperature of 25 °C. The interfacial tension (IFT) of this mixture of biosurfactant and deionized water was then measured. The IFT values were plotted against the corresponding concentrations of the biosurfactant, and the CMC value was measured when the IFT of the increasing biosurfactant concentrations first became minimum and does not decrease further even with the addition of more biosurfactant concentration. The CMC value was expressed as the mass per unit volume of biosurfactant (g/L), following the methodology described by Liu et al.^28^.

#### Functional group determination of biosurfactant by FTIR analysis

The biosurfactant sample to be tested is ground together with anhydrous KBr until homogeneous. Then, the mixture is added to 100 mg of KBr Dye. This mixture is then pressed with a pressure equivalent to 10 tons to form a pellet. The resulting pellet is then inserted into an FTIR instrument for analysis. The infrared (IR) spectrum of the pellets was recorded with a Prestige 21 Shimadzu FTIR spectroscopy instrument. The spectra captured the functional groups present in the biosurfactant, enabling the analysis of its composition. The absorbance spectrum was generated using the instrument’s built-in plotter. The IR spectra were collected within the wave number range of 400–4000 cm^−1^, as described by Das et al.^29^.

### Liquid chromatography mass spectrometry (LCMS) analysis

The LC–MS analysis was conducted in negative ion mode using a Q-TOF Premier model from Waters. The dry biosurfactant was dissolved in an acetonitrile solution and subsequently injected into the LC–MS system, which consisted of a 1260 Infinity LC instrument coupled with a 6410 Triple Quadrupole MS from Agilent Technologies, USA. Mass spectra were acquired within the m/z range of 100–1000, following the procedures described by Goveas et al.^30^ and Sharma et al.^31^. In this study, the LC–MS analysis method employed an MS detector with an ESI ( +) ion source and an MS analyzer in the form of a Q-Tof, enabling the generation of chromatographic peaks. Subsequently, the chromatographic peaks were subjected to analysis using the Masslynx program, as detailed by Sommeng et al.^32^.

#### Stability test of biosurfactant

The Box-Behnken experimental design was selected to investigate the impact of temperature, pH, and salinity on the stability of the biosurfactant activity. The design included three levels for each factor and was conducted in triplicate, resulting in a total of 45 experimental runs, as outlined by Kumar et al.^33^. Both coded and actual variables were utilized and are presented in Table 1. The statistical analysis was performed using MinitabTM 19.0. The main effects and two-factor interactions were estimated through variance analysis (ANOVA), which involved Fisher and Student’s t-tests with a significance level (*α*) of 0.05, following the approach described by Montgomery^34^. The response variable utilized in this study was the emulsification index (EI24) of the biosurfactant. The EI24 was determined after 24 h, serving as an indicator of the biosurfactant’s emulsifying ability.

**Table 1:** Box-Behnken 3^3^ factorial design with actual/coded values and results.

Actual and coded level of factors			E24 (%)
*X*_*1*_	*X*_*2*_	*X*_*3*_	Experimental
40(− 1)	2(− 1)	6(0)	44.08 ± 0.95
120(+ 1)	2(− 1)	6(0)	36.26 ± 0.93
40(− 1)	12(+ 1)	6(0)	7.39 ± 1.08
120(+ 1)	12(+ 1)	6(0)	20.33 ± 2.67
40(− 1)	7(0)	2(− 1)	64.45 ± 3.85
120(+ 1)	7(0)	2(− 1)	55.64 ± 2.19
40(− 1)	7(0)	10(+ 1)	61.71 ± 2.45
120(+ 1)	7(0)	10(+ 1)	43.33 ± 3.34
80(0)	2(− 1)	2(− 1)	24.44 ± 1.93
80(0)	12(+ 1)	2(− 1)	6.91 ± 0.42
80(0)	2(− 1)	10(+ 1)	17.52 ± 1.66
80(0)	12(+ 1)	10(+ 1)	40.88 ± 4.41
80(0)	7(0)	6(0)	61.23 ± 1.09
80(0)	7(0)	6(0)	62.00 ± 1.99
80(0)	7(0)	6(0)	62.00 ± 1.37

X_1_ = temperature (^o^C); X_2_ = pH; X_3_ = Salinity (%).

### Enhanced oil recovery test

#### Sand pack column experiment

The application of biosurfactant in enhanced oil recovery (EOR) was initially assessed using the sand-pack column method, with references to the methodology described by Gudiña et al.^6^ and Astuti et al.^23^ with some modifications. In addition to biosurfactant, the CFS of *P. taiwanensis*, the bacterial cells themselves, and SDS were used in this experiment. As a control, synthetic brine with the same composition as SMSS medium was formulated and used as the brine water in the EOR test.

A glass column with a diameter of 35 mm and a length of 200 mm, equipped with caps on the top and bottom, was packed with quartz sand particles of 50 mesh size. The pore volume (PV) of the column was determined by measuring the amount of synthetic brine water required to fully saturate the column. Subsequently, crude oil was injected into the column until the remaining water reached its saturation point. By measuring the volume of crude oil retained within the column, the original oil in place (OOIP) could be calculated. The initial oil saturation (Soi, %) and initial water saturation (Swi, %) were calculated using the following formulas:

1 $$Soi = \frac{OOIP}{PV}\times 100$$

2 $$Swi = \frac{PV-OOIP}{PV}\times 100$$

Afterwards, the column was subjected to water flooding for a duration equivalent to 2 pore volumes (PV). The volume of crude oil remaining in the column, referred to as the residual oil saturation (Sor), was determined by measuring the displaced crude oil volume. The amount of crude oil mixture recovered after the water flooding, known as the oil-recovered after water flooding (Sorwf, mL), was determined volumetrically. The calculation for the residual oil saturation (Sor) is as follows:

3 $$Sor = \frac{OOIP-Sorwf}{OOIP}\times 100$$

Each column was subjected to flooding with the CFS, crude biosurfactant and bacterial solution respectively. After incubating the column for 24 h, it was once again flooded with water for a duration equivalent to two pore volumes (PV). The effluents from the column were collected to quantify the amount of oil recovered through the use of the crude biosurfactant, referred to as the oil recovered after biosurfactant flooding (Sorbf, mL), which was measured volumetrically. The Additional Oil Recovery (AOR, %) was calculated using the following formula:

4 $$AOR \left(\%\right)= \frac{Sorbf}{OOIP-Sorwf}\times 100$$

The entire process was conducted at a temperature of 50 °C. Control samples, including brine water and SDS at a concentration of 400 ppm, were used for comparison in the enhanced oil recovery (EOR) results. The control column was subjected to identical experimental conditions as the other samples.

#### Core flooding experiment

The application of biosurfactants produced by bacteria in enhancing oil recovery has also been investigated through core flooding experiments. These experiments were conducted at a temperature of 50 °C. The sandstone core utilized in the experiments had a length of 72.17 mm, a diameter of 44.57 mm, a porosity of 13.25% and a permeability of 36.41 mD. On the other hand, the control core had a length of 72.15 mm, a diameter of 44.6 mm, a porosity of 13.61% and a permeability of 43 mD. To determine the pore volume (PV) of the core, the weight difference between the dry core and the core saturated with synthetic brine water was measured under vacuum conditions for 24 h. The synthetic brine water-saturated core was then placed in a core holder with a radial confinement pressure of 1.0 MPa using water.

The core was initially flooded with synthetic brine water at a constant pressure of 0.1 mL/min. Subsequently, crude oil was injected into the core at a pressure of 0.2 mL/min and incubated for 1–3 days to achieve oil saturation. The OOIP was calculated based on the volume of water displaced by the injected crude oil during the oil saturation stage. Primary oil recovery was performed by injecting synthetic brine water into the core for a volume of 2.5 PV, using the same injection procedure as the water flooding stage. The amount of recovered oil was collected at regular time intervals, and the residual oil saturation (Sorwf) was calculated. A volume of 2.5 PV of biosurfactant was then injected into the core at the same injection pressure, and the recovered oil was collected volumetrically as part of the secondary recovery stage (Sorbf). The calculation of additional oil recovery (AOR,%) was performed using the same formula as previously employed in the sand pack column experiment mentioned above. These core flooding experiments and procedures were conducted following previous studies by Joshi et al.^35^ and Purwasena et al.^16^.

## Results and discussions

### Effect of different carbon source on biosurfactant production

Biosurfactants are amphiphilic compounds produced by microorganisms, either on the surface of microbial cells or secreted extracellularly. Their main physiological function is to enable microorganisms to grow on water-insoluble substrates, such as crude oil, by reducing the interfacial tension between the substrate and water. This allows for better absorption and metabolism of the substrate^36^. Biosurfactants can be produced by microorganisms using various carbon sources. The selection of a carbon source is an important step in the production process as different carbon sources can result in variations in biosurfactant structure and production mechanisms. Glucose, glycerol, molasses, and crude oil are commonly used carbon sources for biosurfactant production. Each carbon source has different properties, such as carbon chain length and solubility in water. Microbes can produce biosurfactants on both soluble and insoluble substrates^37^. Different bacterial genera have been reported to utilize different carbon sources for biosurfactant production. For example, *Pseudomonas* can produce biosurfactants better on carbon sources like n-hexane, paraffin, and glycerol, while *Bacillus subtilis* have been reported to preferably utilize waste sunflower oil and cassava flour waste ^38,39^. Furthermore, Jain et al. ^22^ reported that the utilization of starch as a carbon source by *Klebsiella* sp. RJ-03 resulted in the highest production of biosurfactants with better properties.

To evaluate the performance of biosurfactant production from different carbon sources, the interfacial tension and emulsification index of the CFS were measured. The CFS mentioned in this paper contains biosurfactant produced by *P. taiwanensis* and will be referred to as CFS throughout the entire paper. The findings revealed that the lowest interfacial tension and highest emulsification index were observed when crude oil was used as the carbon source. Specifically, the interfacial tension decreased from 15.6 to 14.8 mN/m, 15.6 mN/m, 14.6 mN/m, and 11.9 mN/m when glucose, glycerol, molasses, and crude oil were used as carbon sources, respectively. The emulsification index values were 24.85%, 20.4%, 26.15%, and 59.69% for glucose, glycerol, molasses, and crude oil, respectively, compared to the control with an emulsification index of 8.47%. These results were obtained when comparing the biosurfactant produced in CFS to that of the SMSS medium, which served as the control. *Pseudoxanthomonas taiwanensis* demonstrated its versatility in utilizing a wide range of carbon sources, including both short-chain (glucose) and long-chain (crude oil) carbon compounds with different solubilities in water. The strain exhibited the highest biosurfactant activity when crude oil was used as the carbon source, suggesting its potential as a promising candidate for MEOR applications. Crude oil was then employed as a carbon source to produce biosurfactants for further study in this research.

### Growth profile, biosurfactant yield, Interfacial tension, and emulsification activity

**Figure 1:**
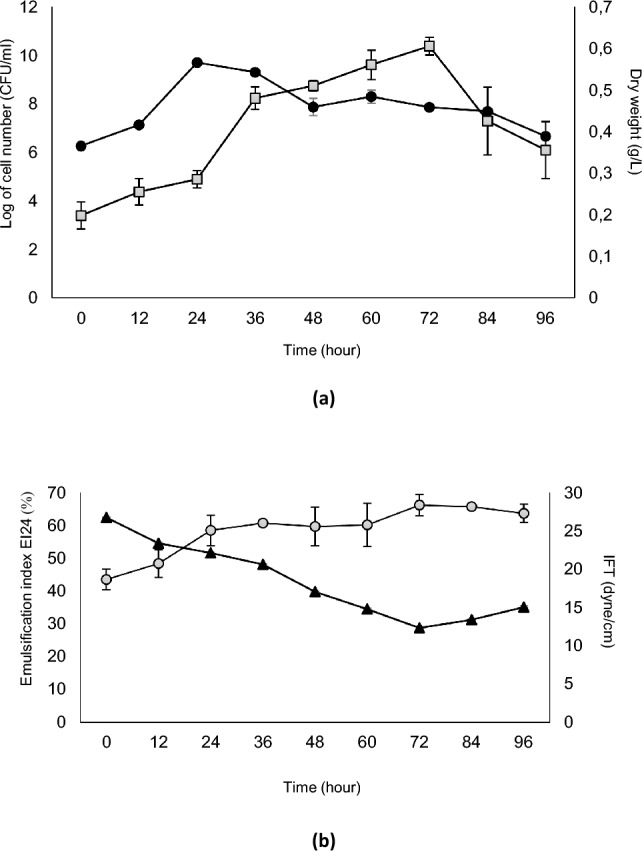
Profile of growth curve and biosurfactant production (**a**) and emulsification index (EI24) and interfacial tension (IFT) (**b**) during the growth of *P. taiwanensis. *(-●- Log of cell number; -□- dry weight of crude biosurfactant**;** -▲-EI24; -○-IFT).

*Pseudoxanthomonas taiwanensis* exhibits a logarithmic growth phase between 12 and 24 h, followed by a stationary phase up to 72 h, and then continue to dead phase until 96 h of incubation time. Figure 1a showed the production of biosurfactant by this strain continues for up to 72 h of incubation and decrease sharply until the end of incubation. This result demonstrated that the highest yield of biosurfactant at 0.6 g/L was produced at the end of stationary phase indicating that the biosurfactant belonged to secondary metabolite. Biosurfactant synthesis is carried out by bacteria to facilitate the uptake of crude oil as a carbon source. The production of biosurfactant by *P. taiwanensis* shows an increase under limiting growth conditions in the stationary phase. Overproduction of biosurfactant occurs when the culture reaches the stationary phase due to limitations in the carbon-to-nitrogen (C/N) ratio^39^. In their review on rhamnolipid production, Reis et al.^40^ revealed that rhamnolipid production increases under environmental stress conditions such as nutrient deficiency, even at low cell density conditions.

The interfacial tension and emulsifying index activity of the CFS were monitored in conjunction with the bacterial growth at 12-h intervals. During the 72-h incubation period, the interfacial tension of the CFS decreased from 26.77 to 12.33 mN/m. Similarly, the emulsifying index activity of the CFS increased as the biosurfactant accumulated in the culture up to 72 h, but declined with further incubation time (Fig. 1b). These findings indicate that *P. taiwanensis* produces and secretes biosurfactants starting from the early stages of growth until 72 h in batch culture using the SMSS medium. Consequently, the biosurfactant produced by *P. taiwanensis* exhibits potential in reducing the interfacial tension between water and crude oil.

### The characteristic of biosurfactant produced by *P. taiwanensis*

The bacteria were cultured in SMSS medium using crude oil as a carbon source to produce biosurfactant. Dilution experiments were conducted to evaluate the effectiveness of the biosurfactant in CFS to reduce interfacial tension (IFT). The IFT values of diluted CFS at different concentrations (100%, 50%, and 10%) were compared to water as a control. The decrease in interfacial tension (IFT) by the supernatant at a 10% concentration is recorded as 3.2 dyne/cm, or a reduction of about 16.49% compared to the control. Meanwhile, at a 100% concentration, the IFT decreases by 6.76 dyne/cm, which is a reduction of about 34.21%. This indicates that increasing the supernatant concentration from 10 to 100% only results in a doubling of the IFT reduction (from 16.49 to 34.21%). These results confirm that the supernatant at a 10% concentration is more effective in reducing IFT compared to a 100% concentration. This implies that the supernatant produced by *P. taiwanensis* is highly effective at lowering IFT even at minimal concentrations, offering benefits for its application in MEOR. Furthermore, using the biosurfactant through direct application of the CFS is more economically viable than employing crude biosurfactant. The comparison of IFT values with a chemically produced surfactant, SDS, indicates that the biosurfactant activity is comparable to SDS at a concentration of 400 ppm. SDS is commonly used in Chemical Enhanced Oil Recovery (CEOR) methods^41^.

Biosurfactant possesses the capability to alter the wettability of rock surfaces by adsorbing at the fluid-rock interface, resulting in a stronger affinity of the rock surface towards a particular liquid, particularly water. Wettability is recognized as a crucial factor in oil recovery^42^ and significantly impacts the interactions between fluids and rocks in multiphase systems. In this study, the effect of wettability was evaluated by measuring the contact angle on glass plates coated with crude oil using water and the CFS. The glass plate coated with crude oil displayed a water-wet system in contact with water (*θ* = 68.0°), while the biosurfactant exhibited an even stronger water-wet system (*θ* = 59.0°) indicating that biosurfactant produced by *P. taiwanensis* capable to increase the water wettability of the surface.

**Figure 2:**
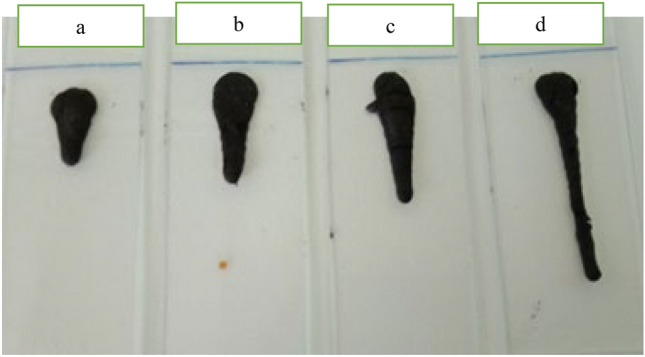
Evaluation of the effect of biosurfactant on a heavy oil (19◦ API) mobility. (**a**) Oil with water = 1.5 cm, (**b**) oil with SMSS medium = 2 cm, (**c**) Oil with CFS = 2.4 cm and (**d**) oil with extract biosurfactant = 3.8 cm.

Biosurfactants offer potential for enhancing the mobilization of heavy oil, including the biosurfactant produced by *P. taiwanensis*. These specialized molecules have the ability to dissolve in heavy oil, reducing their viscosity and facilitating the mobilization of heavy oil fractions^27^. Experimental results demonstrated that the biosurfactant mixed with heavy oil had a spreading distance of 3.8 cm, twice that of water (1.5 cm) as a control (Fig. 2). This suggests that the biosurfactant from *P. taiwanensis* is likely soluble in oil, contributing to its effectiveness in mobilizing and improving the spreading of heavy oil (19° API).

**Figure 3:**
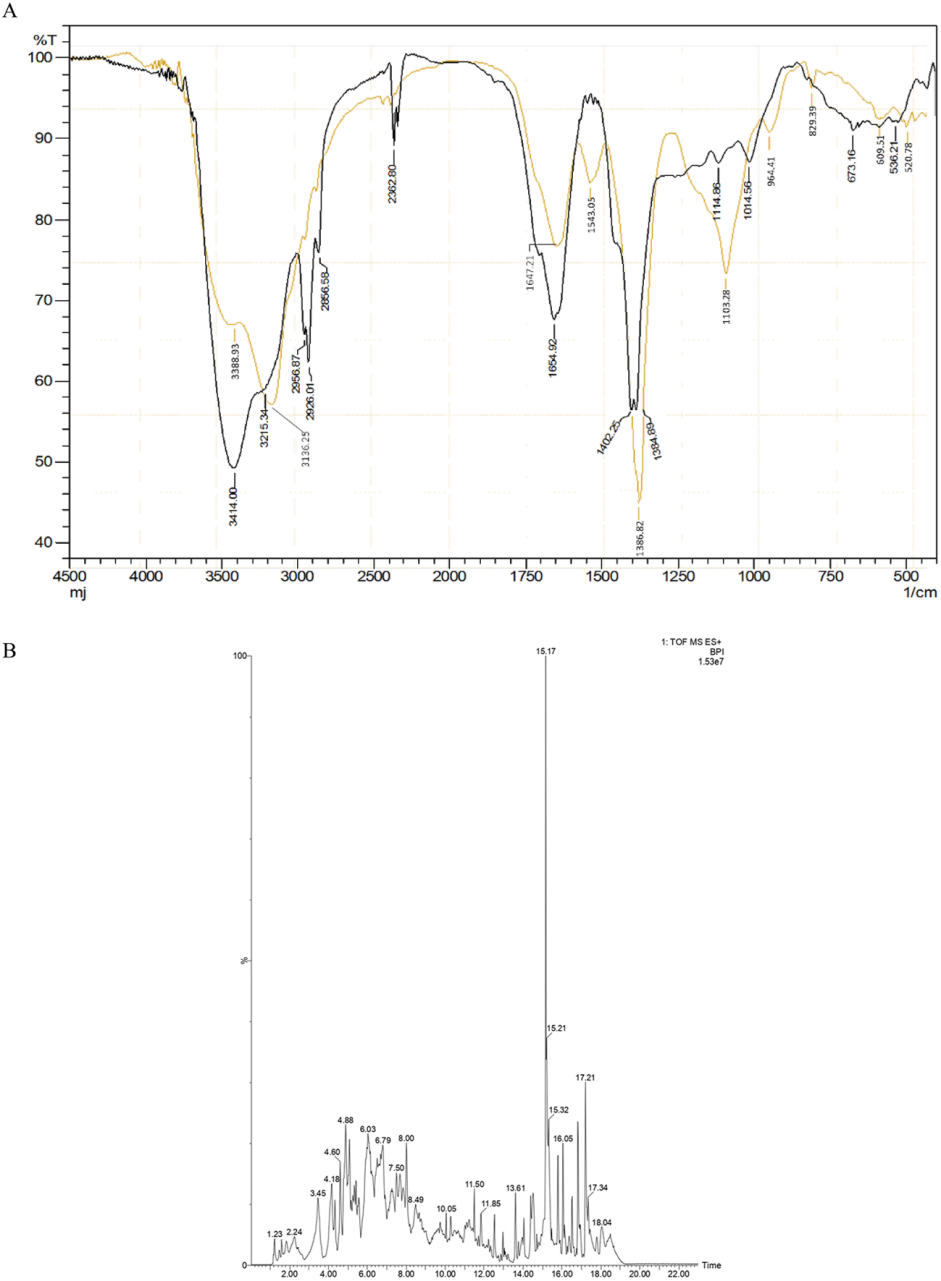
FTIR spectrum (**A**) and LCMS Chromatogram of isolated biosurfactant produced by biosurfactant-producing bacteria *P. taiwanensis* (**B**). Black : biosurfactant produced using light oil obtained from this study; Yellow: biosurfactant produced by using heavy oil (Astuti et al., 2019).

The critical micelle concentration (CMC) is a key parameter that indicates the concentration at which the biosurfactant forms micelles, which are important for reducing interfacial tension and enhancing oil solubility in water^43^. In this study, the CMC point of the biosurfactant-containing CFS produced by *P. taiwanensis* was determined to be 0.33 g/L. It was found that the CMC value of the biosurfactant produced by *P. taiwanensis* using crude oil as a carbon source in this study was lower than the CMC value reported in a previous study using heavy crude oil as the carbon source^23^ where the CMC was determined to be 0.73 g/L. Differences in purity, composition, and variations in growth medium components have been identified as factors that can influence the functional group compositions of the biosurfactant and consequently result in different CMC values, as mentioned by Desai and Banat^36^ and Satpute et al.^44^. The differences in chemical characteristics were further confirmed through its structural analysis (Fig. 3). The CMC value is important as it correlates with the amount of biosurfactant required for efficient oil recovery. Moreover, it should be noted that a significant increase in the biosurfactant concentration will lead to higher production costs.

The IR spectrum analysis of the biosurfactant produced by *P. taiwanensis* reveals the presence of six distinct and sharp peaks (Fig. 3A). The dominant absorption peaks in the IR spectrum correspond to specific functional groups. The peaks observed in the range of 3215–3414 cm^−1^ indicate the stretching of O–H bonds (i), while those in the range of 2926–2956 cm^−1^ represent the stretching of C–H bonds in the aliphatic chain (CH_2_ and CH_3_ groups) of the hydrophobic region of the molecule (ii). The presence of the carboxyl group is indicated by absorption peaks at 2856 cm^−1^ (iii) and 1654 cm^−1^ (iv), corresponding to the O–H and C=O bonds, respectively. The hydrophilic part of the molecule is characterized by the carboxylate group (R–COOH). In the fingerprint region (1500–500 cm^−1^), the IR spectrum shows peaks at 1384–1402 cm^−1^ (v), indicating the presence of CH_2_ and CH_3_ bonds in alkyl groups, and at 1014–1114 cm^−1^ (vi), indicating the C–O stretch in the hydrophilic region of the glycoside groups.

Comparing these findings with the standard rhamnolipid biosurfactant reported by Eraqi et al.^45^, it can be observed that the IR spectrum of the biosurfactant produced by *P. taiwanensis* using light crude oil as a carbon source exhibits similarities, such as the O–H stretch at 3430 cm^−1^, the C–H stretch of CH_2_ and CH_3_ groups at 2938 cm^−1^, the C=O stretch of carboxyl groups at 1629 cm^−1^, and the C–O stretch at 1042 cm^−1^. Based on these results, it can be inferred that the biosurfactant produced by *P. taiwanensis* using crude oil as a carbon source has molecules that are structurally like the glycolipid group. Additionally, the FTIR results of the biosurfactant produced by *P. taiwanensis* in this study exhibit a composition of groups that is nearly identical to the biosurfactant produced by the same strain in previous studies using heavy oil as a carbon source (Fig. 3A). These results agree with the study of which revealed that variation in carbon source did not affect the chemical structure significantly but influenced properties like yield, surface tension, viscosity, emulsification properties, molecular weight, and thermo-stability^22^.

Furthermore, the mass spectrometric analysis of the biosurfactant (Fig. 3B) confirmed the above findings. A total of thirty molecules were detected, seven of which can be categorized as glycolipids, with peak signals observed within the m/z range of 325–669 which are equivalent to their molecular weight. In accordance with Singh et al.^46^, it is noted that the majority of glycolipid biosurfactants possess molecular weights within the range of 302–803 Da. Additionally, a similarity search against the PubChem NCBI database revealed that the LC–MS spectra at m/z 653 and m/z 325 closely correspond to a compound resembling rhamnolipid.

The stability of the biosurfactant produced by *P. taiwanensis* against pH, temperature, and salinity was assessed through the EI24 value, which represents its emulsification activity (Table 1). A polynomial equation (Eq. 5) based on a 2-factor interaction (2FI) model is employed as the simplest numerical model to explain the relationship between controllable variables and the response. This numerical model establishes a correlation between pH, temperature, and salinity, designated as X1, X2, and X3, respectively, with biosurfactant emulsification activity (EI24).

The equation is as follows:

5 $$\begin{aligned} {\text{E24 }} = & { 22}.{7 }{-} \, 0.{137}X_{1} + { 12}.{97}X_{2} + { 1}.{55}X_{3} {-} \, 0.000{19}X_{1} X_{1} {-}{ 1}.{377}X_{2} X_{2} {-} \, 0.{3}0{5}X_{3} X_{3} \\ & + 0.0{\text{259X}}_{{1}} {\text{X}}_{{2}} {-} \, 0.0{\text{132X}}_{{1}} {\text{X}}_{{3}} + \, 0.{\text{511X}}_{{2}} {\text{X}}_{{3}} \\ \end{aligned}$$

This regression model was analyzed using ANOVA to assess its significance and fitness. The statistical analysis reveals that the model is highly significant, confirmed by the Fisher test value (Fmodel = 25.57), which is greater than the tabulated value (F0.05, 9, 35 = 2.16). Thus, with a 95% confidence level in the Fisher *F*-test, the regression model explains a significant portion of the variation in the response values. The regression model exhibits high accuracy, with *R*^2^ and adj-*R*^2^ values of 86.80% and 83.41%, respectively. The significance of each coefficient was determined using the Student’s *t*-test. All parameters, except X3, were found to be significant since their *p*-values are less than 0.05.

**Figure 4:**
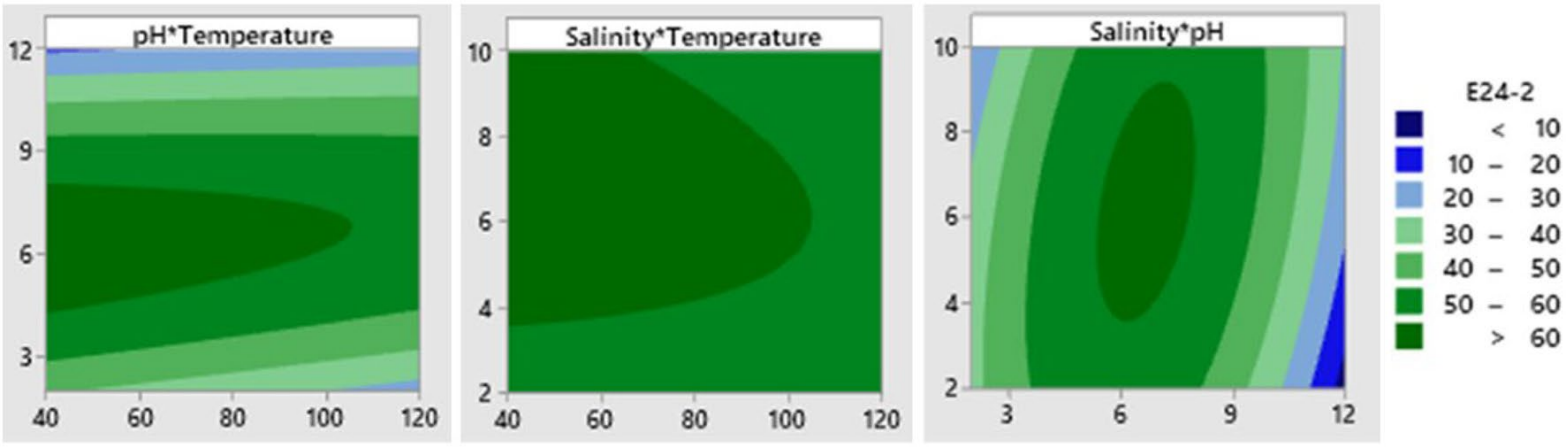
Contour plot of stability test of the biosurfactant produced by *P. taiwanensis* againts environmental factors (pH, temperature and salinity) after 24 h. Hold values: Temperatur 80 °C; pH 7; Salinity 6%.

A two-dimensional contour plot (Fig. 4) was generated to visualize the effect of pH and temperature on the biosurfactant stability, while salinity was held at a fixed level. The results indicated that extreme pH values (< 3 or > 9) led to lower EI24 values, suggesting a decrease in biosurfactant stability. However, temperature and salinity did not significantly affect the biosurfactant stability. It is known that excessively low or high pH can cause the biosurfactant to precipitate and distort its structure, resulting in the loss of its ability to reduce surface tension^47,48^.

The thermostability of the biosurfactant was also evaluated by subjecting the supernatant to high temperatures up to 120 °C. It was found that the biosurfactant performance only slightly decreased, indicating its thermostable nature. Additionally, the biosurfactant demonstrated stable emulsification activity in a wide pH range of 4–10. Furthermore, the addition of NaCl at concentrations ranging from 2 to 10% did not significantly affect the emulsification activity, which remained stable with an EI24 value of more than 40%. These findings align with previous studies that reported the stability of biosurfactants at high temperatures and under varying pH and salinity conditions^16,35,49,50^. Based on the Box Behnken model equation, the predicted optimum values for each variable were determined to be pH 6.4, temperature 40 °C, and 7% salinity, resulting in an EI24 value of 65.21%.

The biosurfactant produced by *P. taiwanensis* demonstrates significant stability and efficacy under optimized conditions (pH 6.4, temperature 40 °C, and 7% salinity), making it a promising candidate for MEOR. Its optimal stability in a neutral pH range, high temperature tolerance up to 120 °C, and resistance to salinity levels between 2 and 10% underscore its versatility for use in diverse reservoir environments. This biosurfactant’s ability to maintain high emulsification activity (EI24 value of 65.21% at optimal conditions) is crucial for enhancing oil recovery by effectively reducing interfacial tension and improving oil mobilization. The adaptability to various pH levels, temperatures, and salinities minimizes the need for stringent environmental control, potentially reducing operational costs and the environmental footprint associated with MEOR operations. Its broad range of stability, combined with the environmental benefits of being biodegradable, positions the *P. taiwanensis* biosurfactant as an environmentally friendly and efficient alternative to synthetic surfactants for improving oil recovery in a variety of reservoir conditions.

Rhamnolipids are often produced by *Pseudomonas aeruginosa* and are well-known for their capacity to lower water surface tension. They are also recognized for their strong emulsifying properties and stability at a variety of pH levels, salt concentrations, and temperatures^51–54^. In contrast, despite their structural similarities to rhamnolipids, *P. taiwanensis* glycolipids are characterized by their higher efficiency at lower concentrations in lowering interfacial tension using CFS, which is critical in MEOR. This efficiency could make them more cost-effective than typical rhamnolipids. They are also more advantageous for MEOR applications due to their capacity to mobilize heavy oils and to increase the wettability of oil-coated surfaces to water.

### Application of biosurfactant in enhancing oil recovery

**Figure 5:**
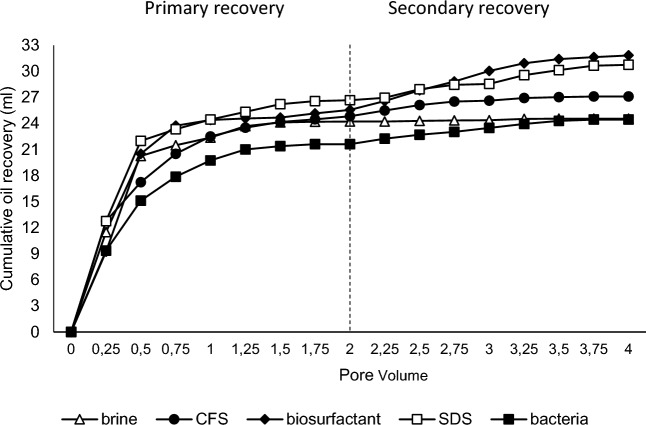
Primary and secondary oil recovery of sand-pack column experiments. SDS concentration: 400 ppm.

The sand-pack column (SPC) is a laboratory-scale testing design that aims to mimic the actual conditions of oil reservoirs in the oil field for studying enhanced oil recovery (EOR) mechanisms^6^. The porosity within the column and the original oil in place (OOIP) showed similar values for all the samples used. This indicates a similar sand structure and permeability zones. The sand-pack column system exhibited porosity ranging from 25 to 29% and OOIP of approximately 45–50 mL. Figure 5 illustrates the amount of oil recovery that occurs during the primary and secondary recovery processes in the column. The oil recovery amount decreases after the brine flooding process of approximately 1.5–2 PV. The residual oil saturation (Sor) in the column was calculated after primary recovery using brine flooding, resulting in values of 42–49%. Subsequently, secondary recovery was conducted using either chemical or bacterial flooding using CFS, biosurfactant extract, *P. taiwanensis* bacterial cells, SDS, and brine water as a control. Additional oil recovery percentages obtained were 9.16%, 36.04%, 10.56%, 15.27%, and 1.33% respectively for CFS, biosurfactant extract, *P. taiwanensis* bacterial cells, SDS, and brine water (Table 2). The use of biosurfactant extract in the secondary recovery phase yielded the highest additional oil recovery (AOR) compared to CSF, bacterial cells and SDS. This indicates that the biosurfactant produced by *P. taiwanensis* is capable to enhance the recovery of residual oil within the column.

**Table 2:** Oil recovery process by sand pack column and core flooding experiments.

Parameter	CFS	Biosurfactant	Bacteria	Control (SDS)*	Control (water)*	Control (water)*	Biosurfactant
	Sand pack column experiment					Core flooding experiment	
PV (mL)	56.00 ± 2.28	58.00 ± 2.00	56.12 ± 2.65	56.50 ± 4.95	53.25 ± 2.47	14.77 ± 0.74	14.90 ± 0.56
Porosity (%)	28.28 ± 1.43	29.29 ± 1.01	28.35 ± 1.34	28.39 ± 2.48	27.21 ± 1.26	13.11 ± 0.65	13.23 ± 0.50
OOIP (mL)	49.32 ± 2.01	50.25 ± 0.75	48.37 ± 1.59	48.50 ± 2.12	43.00 ± 2.82	9.30 ± 1.13	9.45 ± 0.91
Soi (%)	88.08 ± 0.85	86.63 ± 1.70	86.22 ± 1.23	85.84 ± 3.78	80.75 ± 1.56	62.94 ± 4.49	63.42 ± 3.76
Swi (%)	11.92 ± 0.85	13.37 ± 1.70	13.78 ± 1.23	14.15 ± 3.78	19.24 ± 1.56	37.05 ± 4.49	36.57 ± 3.76
Sorwf mL	24.82 ± 3.01	25.47 ± 0.02	21.63 ± 1.23	26.67 ± 0.60	24.52 ± 2.01	5.12 ± 0.69	4.78 ± 1.07
Sor (%)	49.75 ± 4.04	49.30 ± 0.70	55.31 ± 1.09	44.97 ± 1.17	42.99 ± 0.93	44.02 ± 14.26	49.75 ± 6.46
Sorbf (mL)	2.27 ± 0.25	8.77 ± 5.02	2.83 ± 0.03	4.07 ± 0.31	0.32 ± 0.10	0.11 ± 0.05	0.60 ± 0.19
AOR (%)	9.16 ± 1.38	36.04 ± 21.34	10.56 ± 0.27	15.27 ± 2.76	1.33 ± 0.49	2.09 ± 0.07	12.92 ± 3.73

Control*: SDS (sodium dodecyl sulfate); water (artificial brine water).

The use of SPC as a laboratory-scale simulation tool for EOR has been reported to increase the recovery of residual oil from 6.7 to 86%^6,55,56^. However, the oil recovery process within the SPC does not accurately represent the AOR values at the field scale. The SPC is composed of sand that is nearly uniform in type and size, thus it does not fully represent the actual reservoir conditions. Advanced EOR simulation experiments, such as core flooding, can provide a more accurate estimation of the potential oil mobilization by microorganisms or their metabolites^56–58^.

**Figure 6:**
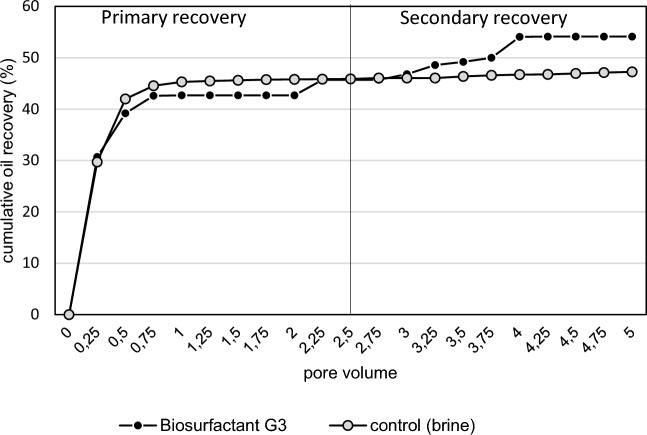
Primary and secondary oil recovery using biosurfactant flooding produced by *P. taiwanensis* on core flooding experiment.

Therefore core flooding experiment was conducted in this study to assess the effectiveness of the biosurfactant produced by *P. taiwanensis* in MEOR. Figure 6 presents the cumulative oil recovery achieved through primary and secondary recovery. Initially, the oil saturation (Soi) in the core was approximately 63–66%, and after injecting 2.5 pore volumes (PV) of brine, the residual oil saturation (Sor) reduced to about 50–54%. Upon initiating brine injection, there was a significant increase in oil production, and stabilization occurred after approximately 0.75 PV. Subsequently, an additional oil recovery was observed after injecting 2.5 PV of the biosurfactant solution, resulting in an AOR of 12.92% over the residual oil in the core, while AOR for control just only 2.09% (Table 2). The findings demonstrate that the biosurfactant produced by *P. taiwanensis* can facilitate the mobilization of crude oil trapped in rock pores. Biosurfactants reduce the interfacial tensions of reservoir fluids, altering the wettability of the rock, thus promoting the release of oil and enhancing oil recovery. These findings highlight the potential of *P. taiwanensis* biosurfactants in MEOR applications. Accurately simulating reservoir conditions, controlling the variability in biosurfactant performance across various oil types and reservoir rocks, and scaling up from laboratory conditions to field applications are potential problems in such investigations. To tackle these obstacles, meticulous experimental planning is frequently required. This includes employing control experiments, modifying experimental settings to more closely mimic field conditions, and carrying out several trials to guarantee the repeatability and reliability of findings. Thus, further testing at the pilot field application is required to fully understand the biosurfactant’s potential and limitation in practical MEOR applications.

## Conclusions

In conclusion, this study highlights the promising potential of biosurfactants, particularly those produced by *P. taiwanensis*, in MEOR processes. The choice of carbon source significantly influenced biosurfactant production, with crude oil proving to be the most effective source. This suggests that this biosurfactant is produced by indigenous bacteria found in oil reservoirs. The biosurfactant exhibited remarkable stability and efficacy under optimized conditions, including a neutral pH, high-temperature tolerance, and resistance to varying salinity levels. Comparisons with sodium dodecyl sulfate (SDS), surfactants commonly used in chemical enhanced oil recovery (CEOR), reveal that the biosurfactant performs comparably or even outperforms SDS in enhancing oil recovery. This highlights its potential as a sustainable and efficient MEOR agent. Furthermore, the biosurfactants demonstrated excellent interfacial tension reduction and emulsification activity, even at low concentrations. Additionally, they were found to enhance the wettability of rock surfaces towards water and effectively mobilize heavy oils, indicating their potential in recovering heavier fractions of crude oil. Laboratory-scale experiments using sand-pack columns and core flooding demonstrated the biosurfactants’ ability to enhance oil recovery, both in porous media and core samples. Overall, the biosurfactants produced by *P. taiwanensis* show promise for MEOR applications due to their efficiency, stability, and versatility under various conditions. However, further research is needed to assess their performance on a larger scale and in field applications. Additionally, factors such as compatibility with different types of reservoir rocks and oils, as well as economic feasibility, should be considered in future research and potential industrial applications.

## Data Availability

The data used and obtained during the study will be available from the corresponding author on reasonable request.
